# Management options for stage 1 nonseminomatous germ cell tumors of the testis

**DOI:** 10.4103/0970-1591.60455

**Published:** 2010

**Authors:** Stephen D. W. Beck

**Affiliations:** Department of Urology, Indiana University, Indianapolis, Indiana, USA

**Keywords:** Retroperitoneal lymph node dissection, stage 1, testis cancer

## Abstract

Management of clinical stage I non seminomatous germ cell tumor includes surveillance, primary chemotherapy and retroperitoneal lymph node dissection. Stratifying clinical stage I disease to high-and low-risk groups for harboring micrometastic retroperitoneal disease (pathologic stage B) is based on pathologic characteristics of the primary tumor. The presence of embryonal dominant histology and lymphovascular invasion (high-risk group) predicts for a 50% incidence of retroperitoneal disease. Low-risk group, the absence of either factor, predicts a 20% chance of retroperitoneal disease. Irrespective of risk classification, all treatment modalities have equal survival rates of 99% to 100%, and differ only in their unique short and long-term modalities. The mode of treatment in clinical stage I disease should remain patient driven and is guided by the perceived morbidities of each therapy.

## INTRODUCTION

Testicular germ cell cancer occurs in 5–7/100,000 men and is the most common solid malignancy in the age group 15–35. Prior to the introduction of cisplatin-based chemotherapy, durable complete remissions of metastatic disease were infrequent, usually less than 15%. It is now established that more than 95% of patients with early-stage testicular cancer and up to 70% of patients with advanced disease will survive. In fact, with cisplatin-based combination chemotherapy, testicular cancer has become a model for a curable neoplasm.

Despite dramatic advances in cure, controversy remains regarding the optimal management of clinical stage (CS) 1 nonseminomatous germ cell tumors (NSGCT), defined as disease limited to the testicle with normal abdominal and chest computed tomographic (CT) scans, and normal serum tumor markers post orchiectomy. The presentation of NSGCT confined clinically to the testicle (CS 1) is associated with a 30–50% incidence of occult retroperitoneal metastases (pathologic Stage 2) creating the controversy regarding “the best” treatment modality. Currently, three approaches are considered for treatment in Stage 1 NSGCT: retroperitoneal lymph node dissection (RPLND), surveillance, and primary chemotherapy, all with equal cure rates at 99%.

The primary issue in the adjuvant treatment of a patient with CS 1 NSGCT is tailoring treatment to those 30% of patients who have occult metastatic disease and are destined to relapse on a surveillance program. Observation without risk assessment will result in the treatment of recurrence in about one-third of patients with multiple courses of chemotherapy and potential resection of residual masses. RPLND as well as adjuvant chemotherapy without risk assessment will over-treat about 70% of patients. It is therefore essential to identify risk factors identifying patients at high risk of occult metastatic disease.

## RISK CLASSIFICATION

The Medical Research Council (MRC) in Great Britain has performed the first major study for identifying risk factors for relapse in CS 1 NSGCT.[1] The multivariate analysis revealed four prognostic factors predictive of recurrence: vascular invasion of the primary tumor, lymphatic invasion, the presence of embryonal carcinoma, and the absence of yolk sac tumor. A prospective MRC trial based on these prognostic variables found the presence of at least three of these four factors to be predictive for relapse in 48% of patients.[2] Vascular invasion was the predominant finding. Conversely, those patients with zero to two risk factors were found to recur on surveillance about 20% of the time.

Multiple other studies have identified similar risk factors for relapse with embryonal cell carcinoma dominant tumors and the presence of lymphovascular invasion consistently being the most powerful predictors. Vergouwe *et al*. performed a review of studies assessing predictors of occult metastases and identified 23 publications reporting on 2,587 patients.[3] Overall 759 (29.3%) patients had occult metastasis. Pooled univariate odds ratios identified lymphovascular invasion (LVI), embryonal carcinoma (EC) >50%, pathologic Stage pT2-4 versus pT1, and MIB-1 staining >70% as the strongest predictors. Though somewhat variable, high-risk groups with the presence of either or both LVI and embryonal dominant primary carry an approximate 50% recurrence rate. Low-risk groups without either pathologic variable had a relapse rate of <20%.

The ability for accurate risk stratification would enable directed therapy: arguably RPLND or primary chemotherapy for the high-risk group and observation for the low-risk group. Even with this stratification, 50% of high-risk patients will be over-treated when otherwise cured with orchiectomy. Likewise, 20% of the low-risk group will be destined to relapse on surveillance and subjected to systemic chemotherapy and possible post-chemotherapy (PC) RPLND.

## SURVEILLANCE

The rationale for surveillance includes 1) the low rate of progression (30% for all comers and 50% for the “high-risk group”) and 2) patients that do relapse remain curable. Irrespective of risk classification, RPLND or immediate chemotherapy, will subject 100% of patients to therapy while arguing benefiting only 30% and up to 50% based on risk classification. That is, even in the high-risk group, 50% of patients are unnecessarily treated with RPLND or chemotherapy.

The group from Toronto reported on 371 patients with CS I NSGCT placed on an active surveillance protocol.[4] The median follow-up was 6.3 years and the median time to relapse was 7.1 months. LVI and pure embryonal cell carcinoma were independent predictors of relapse. In the initial cohort (prior to 1992), 66/157 patients were high-risk and 54.5% relapsed versus 18.7% for the low-risk cohort. In the later cohort (after 1992), 59/214 patients were high-risk and 49.2% recurred versus 14.2% for the low-risk group. In total, 104 (28%) patients relapsed. The disease-specific survival (DSS) was 99.2%.

Similar results were recently published from combined series of 223 patients from British Colombia and Oregon.[5] Fifty-nine (29%) patients relapsed at a median time of four months, 88% relapsed within two years and only seven patients relapsed beyond two years. Treatment at relapse consisted of chemotherapy in 98% of relapses with 78% achieving a complete clinical response. Only 12 of 223 patients (5%) required PC RPLND. DSS was 100% after a median follow-up of 52 months.

## PATTERNS OF RELAPSE

The retroperitoneum (RP) is the most common site of recurrence. On a pooled analysis, Albers reported that approximately 60% of recurrences will be observed in the RP, 25% in the lungs and 10% will be diagnosed based on elevated serum tumor markers (TM) alone.[6] Most recurrences are diagnosed with CT scan or elevated serum TM.

## FOLLOW-UP

Though follow-up schemes vary, it is generally accepted that as the relapse rate is higher in the first two years, follow- up is more intensive during this time period. Schematics should include a combination of physical exam, CXR, serum TM, and abdominal/pelvic CT scan. The NCCN guidelines recommend physical exam, serum TM and CXR every month for Year 1, every other month for Year 2, every three months for Year 3, every four months for Year 4, every six months for Year 5 and then annually. Abdominal/pelvic CT scan is obtained every three months for Year 1, every four months for Years 2 and 3, every six months for Year 5 and then annually. A randomized trial evaluated CT scans at three and 12 months versus three, six, nine, 12, and 24 months and found no benefit in more frequent CT scans. This study involved 414 patients with a median follow-up of 40 months though only 10% of patients were considered high-risk based on vascular invasion.[7]

Arguments against surveillance include compliance and an increased burden of treatment for those patients that do relapse. Those patients that do relapse on surveillance are usually treated with three courses of BEP or four courses of EP with a quarter requiring PC surgery. Patients with RP relapse only with normal serum TM may be considered for primary RPLND. Compliance has been a concern when placing patients on a surveillance protocol with studies showing up to a third of patients missing at least one clinic visit [8–10]

## ADJUVANT CHEMOTHERAPY

The administration of chemotherapy after orchiectomy in CS 1 NSGCT nearly eliminates the risk of relapse. A pooled analysis of 13 studies involving 1043 patients revealed a relapse rate of 1.6% with six patients (0.6%) dying of disease.[11] All but two of these series involved two courses of platinum-based chemotherapy.

With continued data documenting the long-term side-effects of chemotherapy,[12] knowing that 50–70% of CS 1 patients are unnecessarily exposed to chemotherapy (i.e. were never destined to relapse) along with the young population being treated and the fact that other treatment modalities exist with equal cure rates with a lower risk of receiving chemotherapy, this form of management has not gained wide acceptance in the United States.

## PRIMARY RPLND

Retroperitoneal lymph node dissection for CS I nonseminoma has a staging and therapeutic capability. In patients with low-volume RP metastatic disease, surgical cure with RPLND only and without adjuvant chemotherapy occurs at the 65–90% level.[13–16] Indiana University reported on the outcome of 464 patients with CS I NSGCT from 1965–89 with a mean follow-up of 96.2 months.[17] In this analysis, 323 (70%) patients had pathologic Stage A disease with 37 (11%) relapsing, with an overall survival of 99.4%. There were two deaths. Pathologic Stage B disease was identified in the remaining 112 (30%) patients. Of these, 64 did not receive adjuvant chemotherapy, of whom 22 (34%) relapsed with one death. None of the 48 patients receiving adjuvant chemotherapy relapsed.

Recently, the results of RPLND in patients with so-called high-risk, CS I disease treated at Indiana University were reviewed.[18] High risk was defined by the two criteria of embryonal predominance and vascular invasion in the orchiectomy specimen. Embryonal predominance was defined as embryonal carcinoma present at a level greater than any other histologic subtype in the orchiectomy specimen. The presence of each risk factor predicted pathologic Stage B disease at the 46.5% level. Of patients with pathologic Stage B disease who elected not to receive adjuvant chemotherapy only a third had recurrence after RPLND, indicating that two-thirds of these high-risk patients were cured with RPLND only. Therefore, even in so-called high-risk patients, RPLND retains its therapeutic capability. Interestingly, the only identified consequence of primary RPLND in high-risk patients compared to the general population with CS I NSGCT was that those with high-risk features who proved to have pathologic Stage A disease had a recurrence rate of 20% versus 10% in the general population undergoing RPLND.

A contemporary series was recently published from our institution evaluating the efficacy of primary RPLND in patients with pathologic Stage B1 NSGCT. This population included 118 patients, none of whom received adjuvant chemotherapy. At a minimum follow-up of two years, and median follow-up of 43 months, the five-year disease-free survival was 68%. The median follow-up in patients without recurrence was 67.4 months and the median time to recurrence was 5.0 months. Pathologic features including number and histologic subtype of the metastatic lymph nodes failed to predict recurrence.[19,20] Despite the inability to predict risk factors of recurrence in this population, RPLND cures patients with metastatic disease, alone and without adjuvant chemotherapy.

Important in the philosophy of treating low-stage germ-cell cancer is the goal of achieving cure by a single treatment modality. As demonstrated in the studies referenced above, primary RPLND cures 70% of patients with pathologic Stage B disease and the 30% that do relapse remain curable with three courses of chemotherapy. Two courses of adjuvant chemotherapy administered to patients with pathologic Stage B disease after primary RPLND does eliminate the risk of recurrence for those 30% destined to relapse but adds to patient morbidity and unnecessarily exposes chemotherapy to the 70% otherwise cured with surgery.[21,22] If the rationale is to administer postoperative chemotherapy in patients with pathologic B disease in order to avoid recurrence and not rely on surgery for cure, we feel that surveillance is better suited. If this is the case, those patients on surveillance who do relapse would avoid surgery and still be cured with three courses of chemotherapy.

With the introduction of nerve-sparing technique, the morbidity from RPLND is essentially that of a laparotomy.[23–25] A review of the experience at Indiana University showed that the only significant long-term morbidity is an approximate 1% chance of postoperative small bowel obstruction due to adhesions.[26] We recently reviewed the last 75 primary RPLNDs performed at our institution.[27] In this population the mean operative time was 132 min, mean blood loss was 207 cc. We routinely do not place nasogastric (NG) tubes in primary or post-chemotherapy surgery, and in this series only two patients had NG tubes. Clear liquids were started on Day 1 with the mean hospital stay of 2.8 days (range: 2–4). This series demonstrates that in a contemporary cohort the morbidity of open primary RPLND is essentially limited to the incision.

Laparoscopic RPLND (L-RPLND) has emerged as a potential treatment modality in CS 1 disease. Proponents of L-RPLND argue a decrease in morbidity over open RPLND with similar oncologic efficacy. The group from Innsbruck, Austria, has reported the only series of L-RPLND of more than 50 patients. [28] This cohort included 114 CS I patients, after the exclusion of 13 patients because of the learning curve of residents. The mean operative time was 256 min, the mean blood loss was 159 cc (range: 10 to 3000), and the mean hospital stay was 4.1 days. There was one colon and one renal artery injury. A smaller series from the United States, reported the outcome of 29 patients with CS I NSGCT undergoing L-RPLND.[29] The mean operative time was 258 min, and the mean blood loss was 389 cc (range: 75 to 3000). Excluding two open conversions, the mean hospital stay was 2.6 days. This series also reported a mean time to full activity of 17 days after the exclusion of two open conversions. It appears that short-term morbidity, as measured by operative time and hospital stay, is similar for both open[27] and L-RPLND. The only noticeable difference is the significant blood loss of greater than 1 liter observed in the laparoscopic series.[28,29] Inadvertent injury to the aorta, vena cava or lumbar vessels can occur at any time with either approach; however, immediate control and repair of bleeding vessels is more readily accomplished with the open approach as adequate exposure is already obtained.

The oncologic efficacy of L-RPLND has been widely debated as the vast majority of patients found to have pathologic stage 2 disease at L-RPLND receive adjuvant chemotherapy. As such, any potential therapeutic benefit of surgery is unknown as two courses of chemotherapy essentially cure all patients with or without surgery. Investigators from Innsbruck, Austria reported outcomes of L-RPLND in 42 patients of whom 19 had received prior chemotherapy.[30] None of the five patients with pathologic Stage B disease after primary L-RPLND received adjuvant chemotherapy and all are without recurrence at 35, 33, 16, seven, and two months. Prior to proclaiming that L-RPLND is therapeutic and has similar oncologic efficacy as open surgery, the policy of routinely administering adjuvant chemotherapy for pathologic Stage B disease should be addressed.

## OVERVIEW CS 1 NSGCT

The treatment of CS 1 disease should be patient-driven, irrespective of risk grouping as even in the “high-risk group” only 50% harbor micrometastatic disease and the remaining 50% are cured with orchiectomy alone. The advantages and disadvantages of each treatment modality should be discussed along with the perceived short- and long-term morbidity taking into account the uniqueness of each patient and available resources. Future research should be directed towards improved risk categorization.
